# Resoreine

**Published:** 1881-07

**Authors:** 


### RESOREINE.

This new remedy has been found by M. Dujardin Beaumetz to be a benzine, crystallizable, white, odorless, and soluble in all proportions in water. It is an antiferment, and has been employed locally in the treatment of ulcers. Given internally it appears to act like carbolic acid. The Germans employ it in chronic gastritis. The following results have been obtained by its use in cholera infantum :

1. Vomiting has been arrested in a very short time with small doses.
2. The phenomena of collapse diminishes.
3. The stools are less frequent.
4. It is an antizymotic like carbolic acid, but does not irritate nor produce bad symptoms with therapeutical doses as the latter.
5. It is taken willingly ; the stomach tolerates it. Under its influence the stomach quickly regains its lost powers of assimilation. It is given to children under one year in 10-30 centigramme doses. It is necessary to continue its use about six days to obtain a cure.—*Journal de Medicine de Chirurgie Pratiques.*

